# Perspectives of Patients and Care Partners on Benefits or Burdens of Delaying Dementia Progression

**DOI:** 10.1001/jamanetworkopen.2025.17452

**Published:** 2025-06-23

**Authors:** Carolyn Chow, Maayra I. Butt, Jasmine A. Silvestri, Tamar Klaiman, Kristin Harkins, Jason Karlawish, Catherine L. Auriemma

**Affiliations:** 1 Department of Medicine, Perelman School of Medicine, University of Pennsylvania, Philadelphia; 2 Department of Psychology, University of Nevada, Las Vegas; 3 Palliative and Advanced Illness Research Center, Philadelphia, Pennsylvania; 4 Penn Memory Center, University of Pennsylvania, Philadelphia; 5 Division of Geriatrics, University of Pennsylvania, Philadelphia; 6 Leonard Davis Institute of Health Economics, Philadelphia

## Abstract

This qualitative study examines patient and care partner perspectives about the potential of disease-modifying therapies to delay dementia progression.

## Introduction

Recent advances in disease-modifying therapies for persons with mild cognitive impairment (MCI) or mild stage dementia caused by Alzheimer disease (AD) reduce amyloid in the brain and delay progression of functional decline.^1,2^ While experts debate the magnitude and duration of these innovative therapies’ benefits,^3^ patients and families are considering starting treatment. In this new era of AD treatment, physicians must understand if and when disease-modifying therapies are likely to achieve patients’ goals to facilitate shared decision-making. We explored patient and family care partner perspectives on the goals of AD treatment.

## Methods

We conducted semi-structured interviews among patients with MCI or mild stage dementia and family care partners of patients with any stage of dementia. Participants were recruited from a Philadelphia memory center, within a larger mixed-methods study from May 2022 to January 2023.^4^ Dementia stage was defined using the Montreal Cognitive Assessment (MoCA); MoCA-Blind, or Function Rating Scale.^4^ A multidisciplinary research team developed and piloted an interview guide. Box shows the questions analyzed in this report. Video-conferencing or telephone interviews were audio-recorded and transcribed. A codebook was created inductively. The first 17 transcripts were double-coded. During regular consensus meetings, coding disagreements were reviewed and arbitrated. Remaining interviews were divided and coded independently, with every fifth transcript double-coded. Data were analyzed by constant comparision.^5^ Thematic saturation informed sample size. This qualitative study was approved by the institutional review board at the University of Pennsylvania. Participants provided verbal informed consent. This report follows Consolidated Criteria for Reporting Qualitative Research (COREQ) reporting guideline.

Box. **Interview Questions About Treatment Outcomes and Decision-Making**

**Objective:** *Identify the outcomes of dementia care that matter most to participants or would most influence their decision making.*

**Care Partner Version**

When you think about the future for [patient], what do you think matters most to [patient]?
What matters most to you?
Imagine a new treatment was to come out for [patient’s] memory problems. What would you hope it might achieve?

**Patient Version**

When you think about the future, what matters most to you?
Imagine a new treatment was to come out for memory problems. What would you hope it might achieve?

## Results

We interviewed 14 patients and 36 care partners. Approximately half of care partners had loved ones with MCI or mild-stage dementia (19 [53%]) and half with moderate-to-severe stage dementia (17 [47%]). Care partners were spouses (21 [58%]), children (13 [36%]), or siblings (2 [5%]).

We identified several themes (Table). Participants wished for future AD therapies to reverse or cure disease. However, these hopes were often felt to be unrealistic. Slowing or halting disease progression was acceptable for some, but primarily among those whose clinical scores suggested preserved cognitive function. Other care partners remarked that halting or slowing disease progression could in fact be detrimental, causing unintended harms by prolonging or exacerbating patient morbidity and caregiver distress.

**Table.** Themes and Representative Quotations^a^

Theme	Representative quotes	Participant
Participants wish that dementia therapies could be curative, but believe that to be currently unrealistic	I wish there was something that we could do to even end it or make it better or reverse it or something, but there’s nothing really right now that’s available that will do that.	Care partner, spouse of person with moderate dementia
	[I wish] I wouldn’t have the memory problems, that it would ameliorate that, that it would be gone, I would be without that.	Person with mild dementia
	Being able to prevent, because you’re sure not gonna rebuild what’s lost.	Person with mild dementia
	So, it’s—will there ever be anything for Alzheimer? Who knows? That could say, take this and you’ll be fine? I doubt it.	Care partner, spouse of person with MCI
	I mean, realistically, I don’t think they’re gonna fix this. I mean if they could, yay, but I don’t see that happening.	Care partner, spouse of person with moderate dementia
Slowing or halting cognitive decline is desirable primarily for persons with mild stage disease	Well, if I could see that I was plateauing out, it would be wonderful.	Person with MCI
	But if it could stop the progression and allow her to continually live and age in place at this level before it gets too far along.	Care partner, child of person with mild dementia
	Stabilizing where he is right now, stopping progression. That would probably be the biggest gift.	Care partner, spouse of person with mild dementia
	You know, when I think back to where we were 2 years ago, if we could have even just halted it there, that would have been lovely. So I would say anything that would halt progression would be amazing. Anything that, obviously, could turn it around would be even more amazing, but that seems a lot to ask of a drug.	Care partner, child of person with moderate dementia
Slowing disease progression is undesirable for some, and may be perceived as harmful	Well, I hate to be Debbie Downer, but if it would keep him the same as what he is, I wouldn’t let him have it…If it’s not gonna change you and make you better and back to how you were before you had it, then it’s wasted on him.	Care partner, spouse of person with moderate dementia
	If you would have asked me this 2 years ago, I would have said just stop it at that point. You know, you don’t have to bring all his memories back because you can—you could make new ones at that point. But today I don’t think there’s any there, so today if it happened, I would want it to be able to reverse it.	Care partner, spouse of person with severe dementia
	Well, pie in the sky. Reverse it. Ain’t gonna happen ever. Well, if there’s something that could—do I even wanna slow it down? That’s such a hard question. Do I want to?…I don’t know.	Care partner, spouse of person with mild dementia
	I’m almost getting to a point—this is gonna sound really cruel and mean, but if we’re going at the pace we’re going in another year. I don’t know that I want it to get better. How long are we gonna drag this out? I mean, I don’t know how long I could do it.	Care partner, spouse of a patient with moderate dementia

Abbreviation: MCI, mild cognitive impairment.

^a^ Dementia severity was defined using the following thresholds: Montreal Cognitive Assessment (MoCA): MCI, >25; mild, 18-25; moderate, 10-17; severe, <10; MoCA-Blind: MCI, >18; mild, 13-18; moderate, 7-12; severe, <7; mini-mental status examination: MCI, >25; mild, 20-25; moderate, 12-19; severe, <12; Function Rating Scale: mild, <19; moderate, 19-36; severe, >36.

## Discussion

These findings suggest that persons with dementia and family care partners are hopeful for, yet skeptical of, the curative potential of future AD therapies. Stakeholders value therapies that could slow or halt disease progression but only in more mild-stage dementia. Participants’ statements suggest that for some families, such an outcome might be harmful. This was not exclusive to individuals with severe-stage dementia as measured by common clinical scales. These findings have 2 important implications. First, there was a need for continued advancement in more effective disease-modifying treatments. Second, there were aspects of the dementia experience that may cause people to reject slowing the disease even at its earlier stages.^6^ Common clinical scales may not fully capture variables^6^ that impact a family’s impressions of the benefit of delaying dementia progression. Additional research and individualized conversations are needed to understand the appropriateness and timing of existing treatments.

This study has limitations. By enrolling from a single memory center, study findings may not generalize to individuals not cared for in memory centers or from different regions. We used thematic saturation to determine sample size. Alternative methods exist.

In the course of dementia, there may be an inflection point at which the morbidity outweighs the potential benefit of slowing dementia progression. As access to AD disease-modifying therapies increases, clinicians must prepare to have individualized conversations with patients and families about when to consider starting therapy and when to stop.

## Notes

Supplement.
Data Sharing Statement
