# Supplementary material for: Perspectives of Patients and Care Partners on Benefits or Burdens of Delaying Dementia Progression
Source: JAMA Netw Open. 2025 Jun 23;8(6):e2517452. doi: 10.1001/jamanetworkopen.2025.17452 (PMC12186506; doi:10.1001/jamanetworkopen.2025.17452)
Supplement: Supplement. — Data Sharing Statement [file jamanetwopen-e2517452-s001.pdf]

# Data Sharing Statement

Chow. Perspectives of Patients and Care Partners on Benefits or Burdens of Delaying Dementia Progression. *JAMA Netw Open*. Published June 23, 2025.
doi:10.1001/jamanetworkopen.2025.17452

**Data**
**Data available:** Yes
**Data types:** Deidentified participant data
**How to access data:** Catherine.auriemma@pennmedicine.upenn.edu
**When available:** beginning date: 01-01-2026

**Supporting Documents**
**Document types:** None

**Additional Information**
**Who can access the data:** researchers whose proposed use of the data has been approved
**Types of analyses:** for a specified, approved research proposal
**Mechanisms of data availability:** after approval of a proposal and with a signed data access agreement
